# A dataset of the thioacetmide supported formation of ZrO_2_ coating on Ni-rich layered structure cathode materials in lithium-ion batteries

**DOI:** 10.1016/j.dib.2020.105458

**Published:** 2020-03-20

**Authors:** Van-Chuong Ho, Seunghun Jeong, Taeeun Yim, Junyoung Mun

**Affiliations:** a Department of Energy and Chemical Engineering, Incheon National University, 12-1, Songdo-dong, Yeonsu-gu, Incheon 22012, Republic of Korea; b Department of Chemistry, Research Institute of Basic, College of Science, Incheon National University, 12-1, Songdo-dong, Yeonsu-gu, Incheon 22012, Republic of Korea

**Keywords:** ZrO_2_ Coating, Thioacetamide (TA) hioacetamide (TA), Ni-rich layered cathode, pH of the coating solution

## Abstract

A dataset in this report is regarding a research article “Crucial Role of Thioacetamide for ZrO_2_ Coating on the Fragile Surface of Ni-rich Layered Cathode in Lithium Ion Batteries” [1]. Thioacetamide (TA) is introduced to form a homogeneous ZrO_2_-coating in a facile method through washing with Zr(SO_4_)_2_ aqueous solution. The presence of the data in this paper indicated the role of TA for surface modification of LiNi_0.82_Co_0.09_Mn_0.09_O_2_ (NCM82) materials by ZrO_2_, leading to improve the electrochemical performance of NCM82 Ni-rich cathode materials. These data were proceeded measurement electrochemical properties of cathode electrode on a battery cycler, the surface characteristics of the cathode materials were investigated by SEM, EDS mapping, TEM and XPS. X-ray diffraction (XRD, Rigaku, SmartLab) was used to evaluate the influence of the coating layer on the microstructure of active materials.

**Specifications table**

**Table:** 

Subject	Energy
Specific subject area	Renewable Energy, Sustainability and the Environment
Type of data	Figure
	Image
How data were acquired	XRD, XPS, SEM, TEM, Lithium impurities, pH value, Voltage profiles, cycles and rate performance.
Data format	Raw and Analyzed
Parameters for data collection	pH value, the thickness of the ZrO_2_ coating layer, applied current and voltage, cycle at room temperature and 45 °C and rate performance.
Description of data collection	Surface morphologies, pH condition, homogeneous coating, cycle and rate performance of the cells influenced by ZrO_2_ coating layer on the cathode electrode.
Data source location	Center for UI Research Facilities, Incheon National University, 12–1, Songdo-dong, Yeonsu-gu, Incheon, Republic of Korea.
	LAB, Department of Energy and Chemical Engineering, Incheon National University, 12–1, Songdo-dong, Yeonsu-gu, Incheon, Republic of Korea.
	Department of Chemistry, Research Institute of Basic, College of Science, Incheon National University, 12–1, Songdo-dong, Yeonsu-gu, Incheon, Republic of Korea.
Data accessibility	The data are available with this article.
Related research article	Van-Chuong Ho, Seunghun Jeong, Taeeun Yim, Junyoung Mun, “Crucial Role of Thioacetamide for ZrO_2_ Coating on the Fragile Surface of Ni-rich Layered Cathode in Lithium Ion Batteries”, J. Power Sources, https://doi.org/10.1016/j.jpowsour.2019.227625

## Value of the Data

• This research is essentially understanding the role of TA in the surface modification for Ni-rich cathode materials by ZrO_2_ via washing in Zr(SO_4_)_2_ aqueous solution.
• Data in this paper shows a homogeneous ZrO_2_ layer formed on the surface of NCM82, leading to enhance the electrochemical performance of Ni-rich cathode materials.
• In this work, the TA has been controlling the pH value of the coating solution and supported Zr ions evenly distributed on the surface of Ni-rich cathode materials.
• This data provides a simple surface modification method for NCM Ni-rich cathode materials, low cost with ZrO_2_ coated layer-based Zr-precursor and can be widely applied for coating technologies of transition metal oxide.

## 1 Data description

**Fig. 1:**
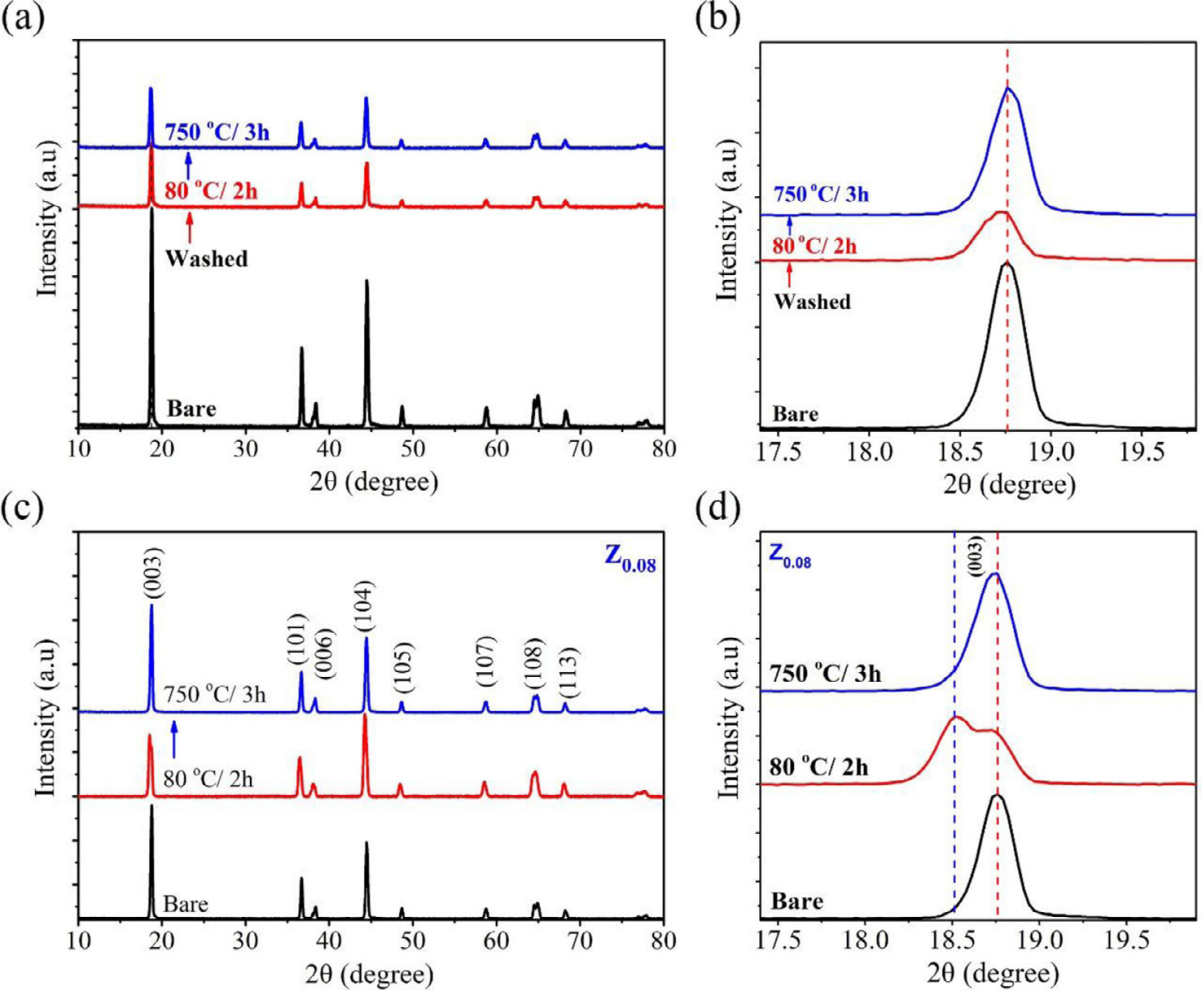
Effect of washing process on the structure of NCM82 Ni-rich materials. (a), (b) are washed sample and (c), (d) are ZrO_2_-coated NCM82 Ni-rich by 0.08 M Zr(SO_4_)_2_/ D.I water solution.

**Fig. 2:**
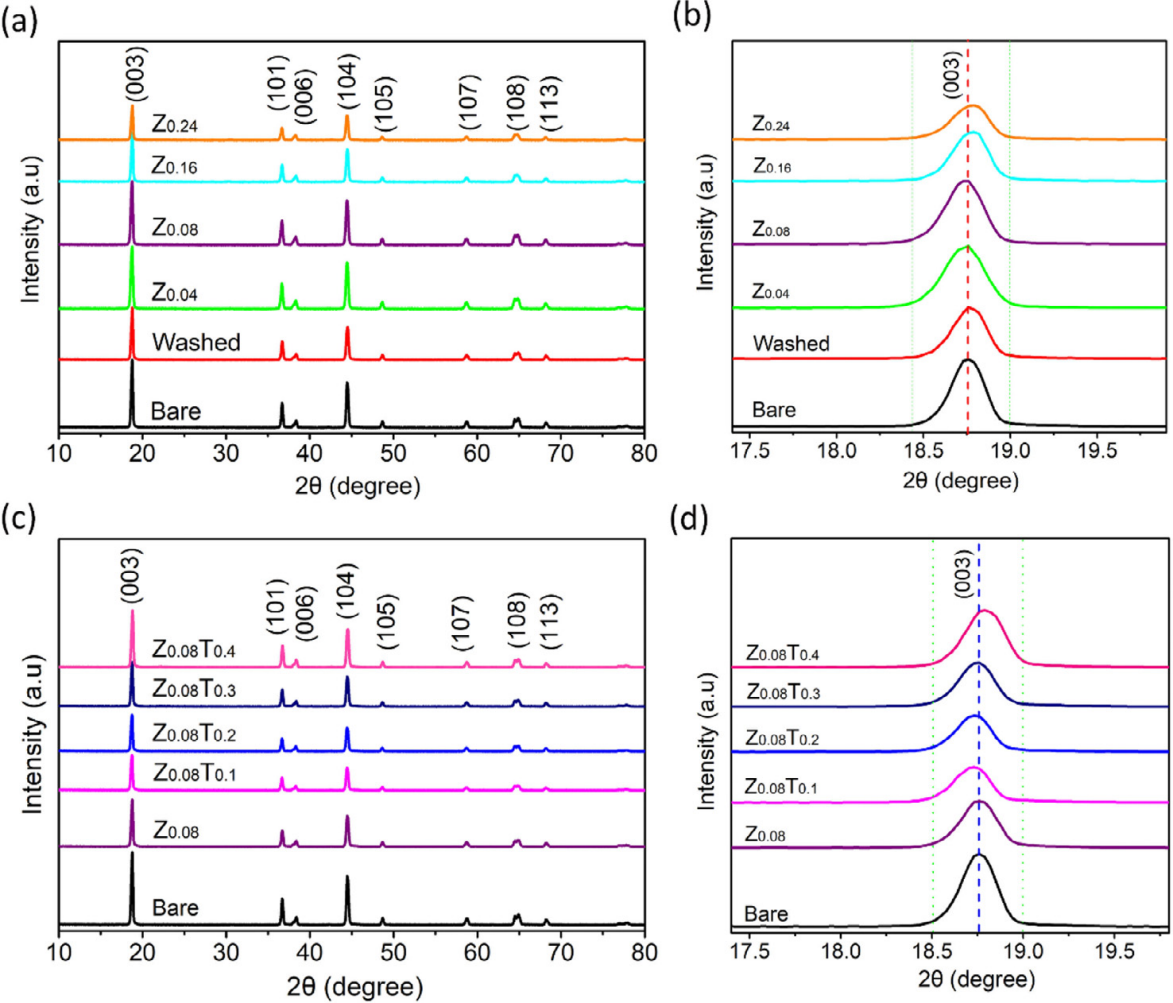
(a), (b) XRD spectra of the ZrO_2_-coated NCM82 using a different concentration of Zr(SO_4_)_2_ without TA. (c), (d) XRD spectra of the ZrO_2_-coated NCM82 using 0.08 M Zr(SO_4_)_2_ with different concentration of TA varying from 0.1 to 0.4 M.

**Fig. 3:**
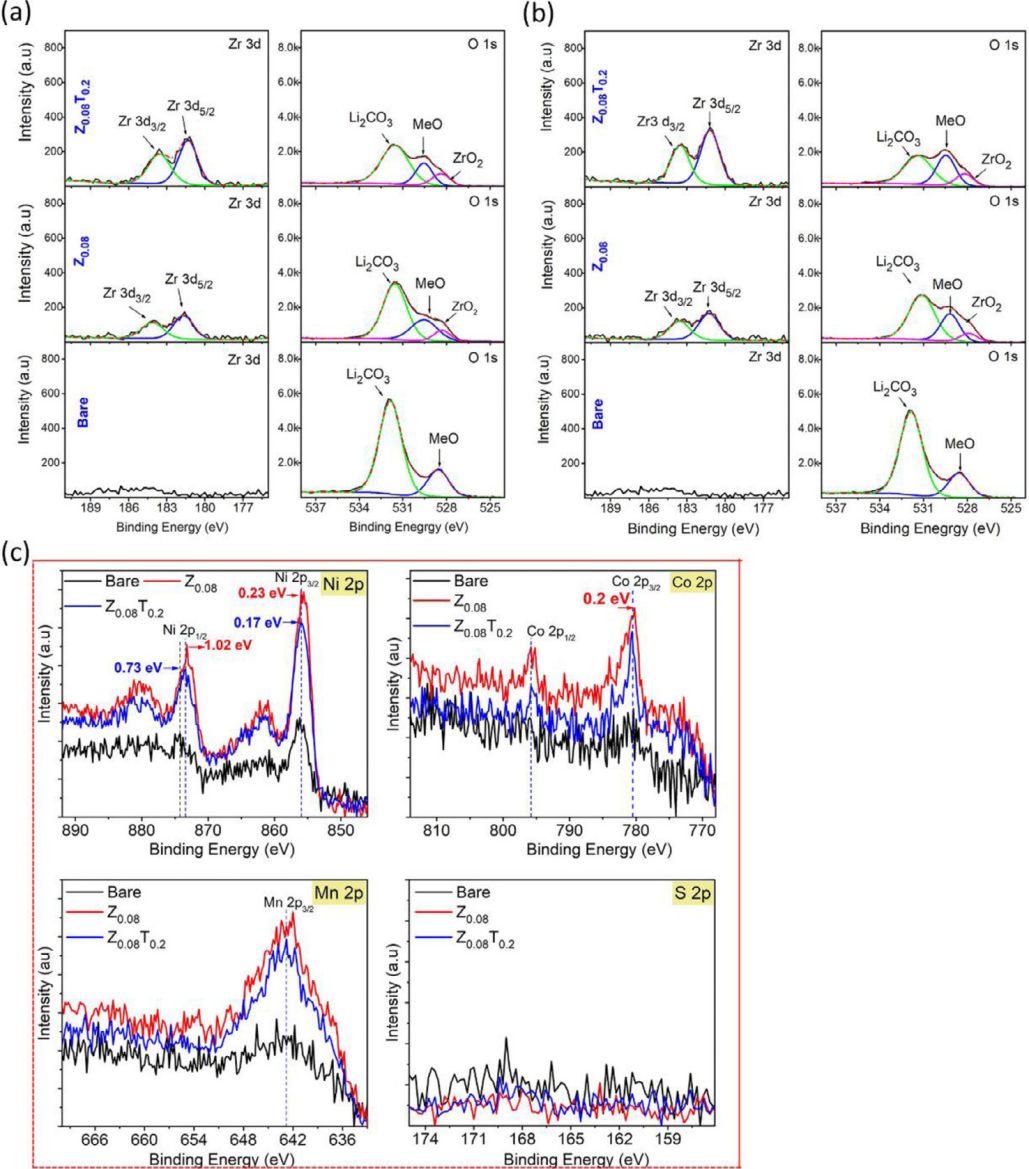
The XPS patterns of the ZrO_2_-coated NCM82 Ni-rich material was calcinated at 750 °C for 3 h in oxygen. In this experiment, 0.08 M Zr(SO_4_)_2_ with 0.2 M TA and without TA was used as a coating solution. (a) after Ar-etching 3 s, (b) after Ar-etching 30 s and (c) XPS peaks of Ni 2p, Co 2p, Mn 2p and S 2p of bare, Z_0.08_ and Z_0.08_T_0.2_ without Ar-etching, (d) the fitting Ni 2p, Co 2p, Mn 2p XPS spectra of bare, Z_0.08_ and Z_0.08_T_0.2_ without Ar-etching.

**Fig. 4:**
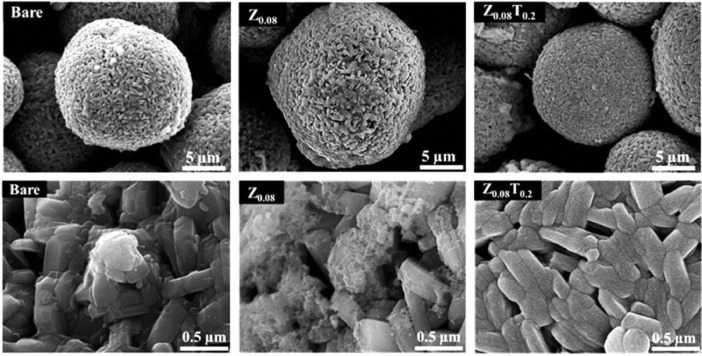
SEM images show morphologies of the bare NCM82, Z_0.08_ and Z_0.08_T_0.2_ samples.

**Fig. 5:**
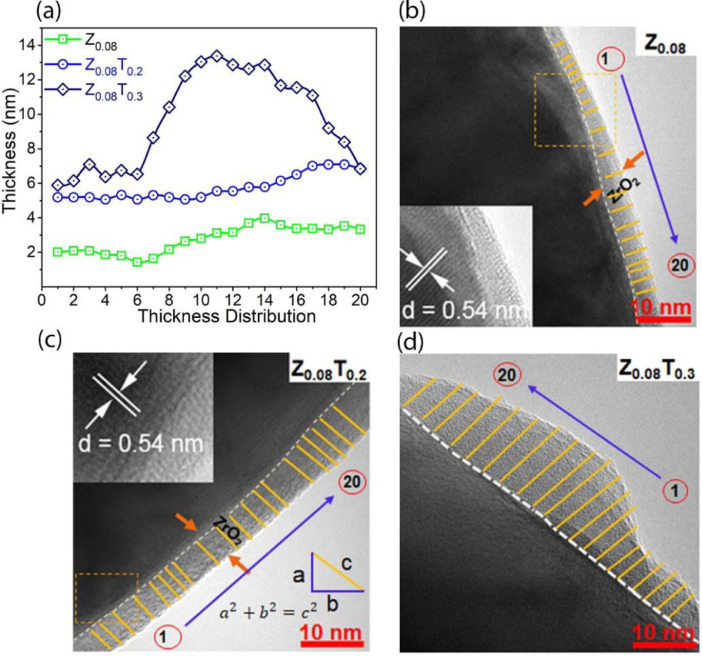
The thickness of the ZrO_2_ coating layer was carefully determined by ImageJ software and simple excel method. (a) the thickness distribution forms on the surface of the Z_0.08_ and Z_0.08_T_0.2_ sample, (b) TEM image of Z_0.08_, (c) TEM image of Z_0.08_T_0.2_ and (d) TEM image of Z_0.08_T_0.3_.

**Fig. 6:**
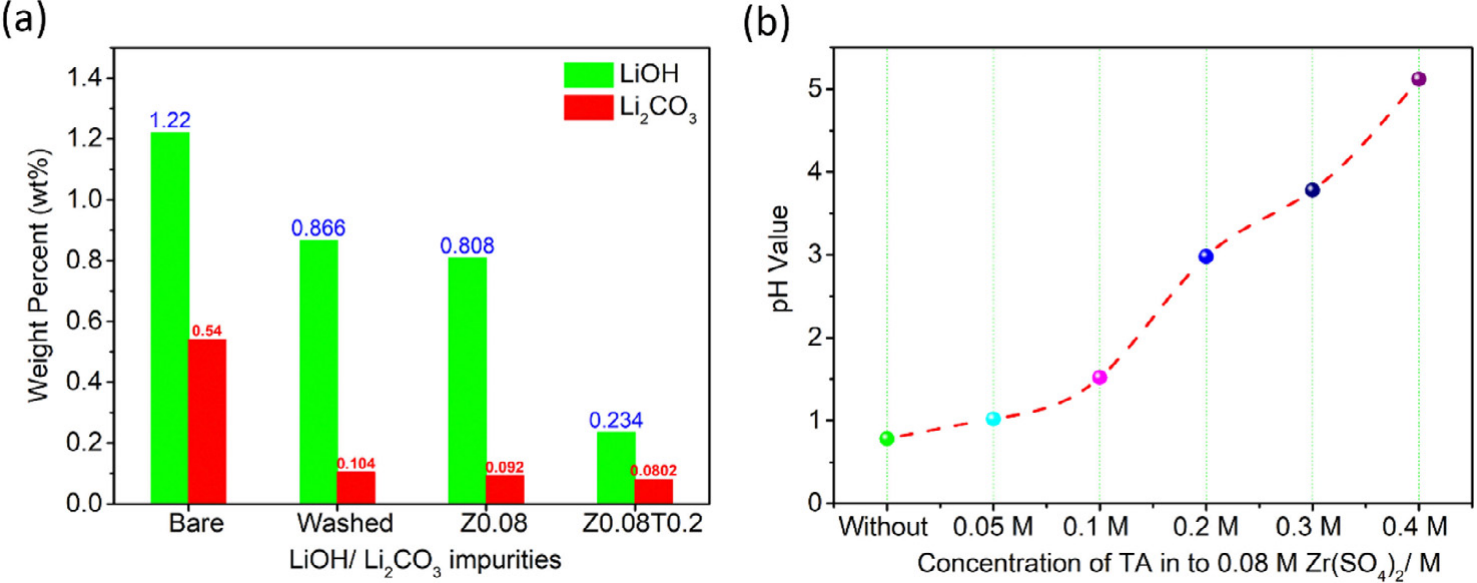
(a) Lithium impurities were performed measurement by titration method on Titrino Plus 848 machine, (b) pH value of coating solution as a function of TA concentration.

**Fig. 7:**
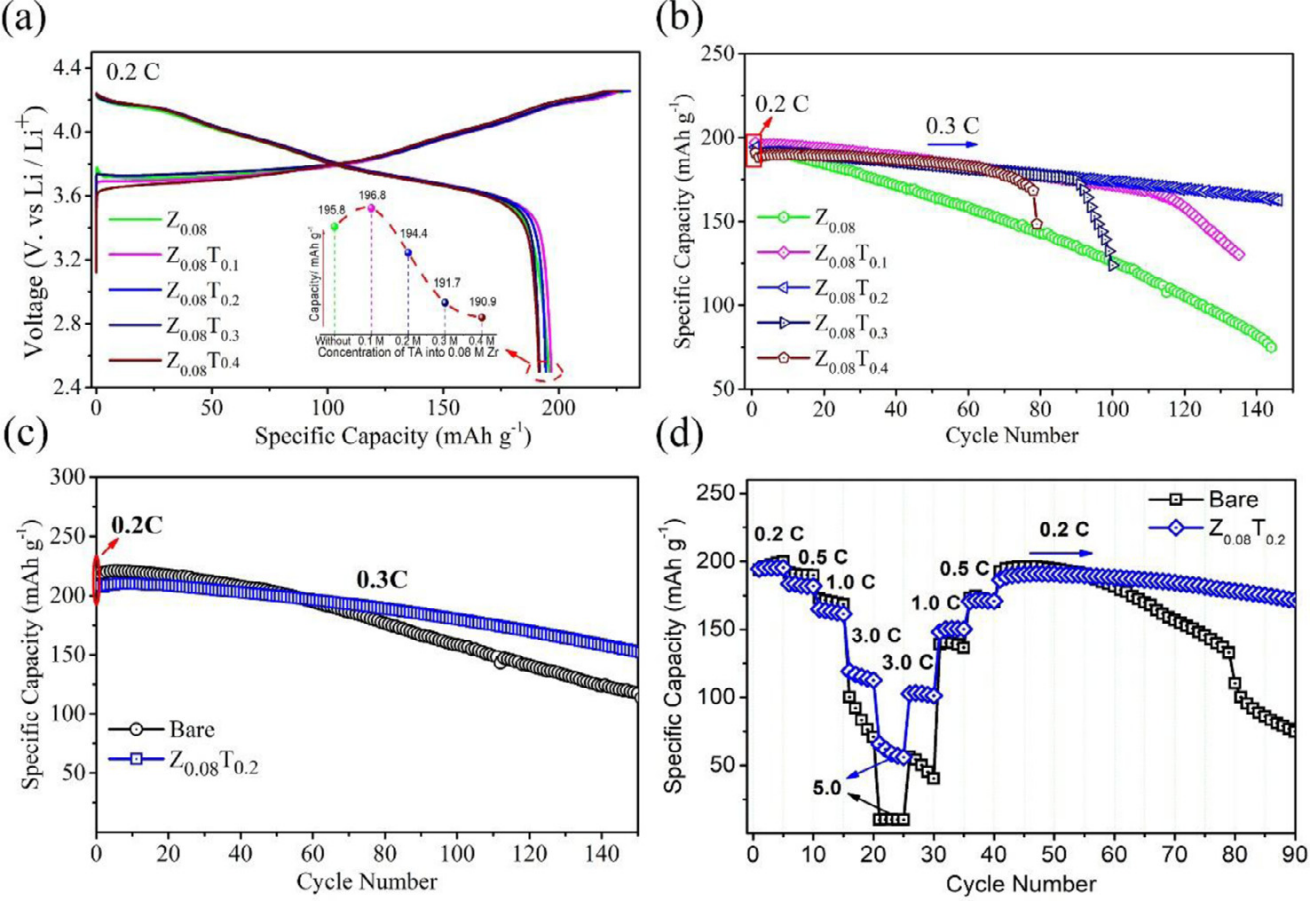
Effect of concentration of TA on the electrochemical performance of the ZrO_2_-coated NCM82 Ni-rich cathode materials. (a) initial charge/ discharge voltage profiles at 25 °C, (b) specific discharge capacity at 25 °C, (c) specific discharge capacity of bare NCM82 and Z_0.08_T_0.2_ sample at 45 °C and (d) rate performance of the bare NCM82 and Z_0.08_T_0.02_ at 25 °C. The half-cells were cycled at 0.3 C after formation cycles 0.2 C and a voltage of 2.5–4.25, (1 C = 200 mA *g* ^−^ ^1^).

The data in this work has been derived by fabrication of the ZrO_2_-coated layer by simple washing method. Figs. 1 and 2 show the structure change of host materials due to immersed in the coating solution. Fig. 3 shows the effect of different coating solution on the surface coating properties of the NCM82 cathode materials. Figs. 4 and 5 show the effect of coating solution on the surface morphologies of NCM82 materials, simultaneously description the thickness of ZrO_2_ coating layer. Fig. 6 shows the amount of lithium impurities eliminated by coating solution and pH value of the coating solution was controlled by TA. Fig. 7 shows the effect of concentration of TA on the electrochemical performance of the ZrO_2_-coated NCM82 Ni-rich cathode materials.

## 2 Experimental design, materials, and methods

The washing process and simultaneously surface coating by the aqueous solution is the simple method, the impurities on the surface of host materials are elimination and immediately Zr-precipitation to establish a solid protective layer for cathode materials. Many chemical and physical parameters influence the overall process outcome. These include material parameter (pH value, Li delithiation, concentration, thickness and lattice parameter), electrochemical process (fading capacity, cycle and capacity). Experiment detail for the fabrication of ZrO_2_-coated layer has been previously presented. The Zr(SO_4_)_2._•4H_2_O, TA were dissolved in D. I water and NCM82 powders were added in the coating solution, stirred, filtered, dried filtered powders and calcination of black powder materials.

### 2.1 The effect of washing process on the structure change of NCM82 Ni-rich materials

The XRD data of all samples were performed measurement on the X-ray diffraction (XRD, Rigaku, SmartLab) with 2θ in a range from 10 to 80° and scanning mode of 0.3, (Cu target of 1.5412 Å). Fig. 1 shows the XRD spectra of the bare NCM82, NCM82 materials were washed in D. I water, dried at 80 °C for 2 h (NCM-W80) and heated at 750 °C for 3 h (NCM-W750), ZrO_2_-coated NCM82 materials from Zr(SO_4_)_2_ solution without TA was dried at 80 °C for 2 h (Z_0.08_-80) and heated at 750 °C for 3 h (Z_0.08_-750). Fig. 1(a, b) show the XRD pattern of NCM-W80 and NCM-W750 compared with pristine NCM82 sample, observed that the peak at (003) of NCM-W80 shifts to lower 2θ (degree of shift peak, ∆(2*θ*) is 0.04°). Fig. 1(c, d) shows the XRD pattern of the Z_0.08_-80 and Z_0.08_-750, observed that peak at (003) of Z_0.08_-80 had two 2*θ* peaks at 18.74° and 18.53°. The peak at (003) of the NCM-W750 and Z_0.08_-750 shift to a higher angle close to the original peak position of the pristine sample, maybe due to the oxygen defects established in the octahedral structure of NiO_6_ [2,3].

### 2.2 The effects of TA concentration of precursor chemical in coating solution on the structure change of the NCM82 Ni-rich materials

Fig. 2 shows the XRD properties dependence on the concentration of precursor chemical in the coating solution, Fig. 2(a, b) shown the XRD pattern of the NCM82 powders were immersed in the different concentration of Zr(SO_4_)_2_ of 0,04, 0.08, 0.16 and 0.24 M for 5 min, after filtered, dried and calcinated, the samples were named Z_0.04_, Z_0.08_, Z_0.16_ and Z_0.24_, respectively. The peak at (003) is moved to the higher angle 2θ when increases concentration of Zr in the coating solution, due to the amount of Zr-coated increased. The Fig. 2(c, d) show the XRD spectra of coating sample from 0.08 M Zr(SO_4_)_2_ with different TA concentration of 0, 0.1, 0.2, 0.3 and 0.4 M compared pristine sample, the samples were named Z_0.08_, Z_0.08_T_0.1_, Z_0.08_T_0.2_, Z_0.08_T_0.3_ and Z_0.08_T_0.4_, respectively. The peak at (003) is shifted to the higher angle 2θ when increases concentration of TA in the coating solution, TA could be agent controlling thickness of ZrO_2_-layers as well as the amount of Zr-precipitation on the host materials.

### 2.3 The characterization of surface modification for Ni-rich layered oxide cathode materials

The chemical composition on the surface of the pristine NCM82, Z_0.08_ and Z_0.08_T_0.2_ were determined by XPS analysis as shown in Fig. 3, all XPS spectra were calibrated against the C 1 s binding energy at 285 eV. The XPS measurement has proceeded after Ar-etching 3 s (Fig. 3(a)) or 30 s (Fig. 3(b)). Z_0.08_ and Z_0.08_T_0.2_ show the characterization peaks at 184.10 eV (Zr 3d_3/2_) and 181. 68 (Zr 3d_5/2_), which was observed in the Z 3d spectra, and intensity of the Zr 3d_3/2_ and Zr 3d_5/2_ peaks with Z_0.08_T_0.2_ is much higher than that these peaks in Z_0.08_ sample at the same condition [1]. The one of the characterization of lithium impurity (Li_2_CO_3_) was observed in O 1s spectrum (at 531.8 eV), the intensity of this peak in is decreasing for Z_0.08_ and Z_0.08_T_0.2_ compared with the bare sample.

### 2.4 Surface morphologies analysis of bare sample, Z_0.08_ and Z_0.08_T_0.2_

Surface morphologies of the bare NCM82, Z_0.08_ and Z_0.08_T_0.2_ were shown in Fig. 4(a), the ZrO_2_ coating layer on the surface of Z_0.08_T_0.2_ is relatively uniformity, meanwhile, a coating materials layer on the surface of Z_0.08_ is un-even distributed. For the bare sample, the white grain was observed that is the lithium impurities as a report of X. Xiong et al. [3]. In addition, Zr element is more evenly distributed on the surface of the Z_0.08_T_0.2_ sample compared with bare sample [1].

### 2.5 Effect of coating solution on the distribution ZrO_2_-thick coating layer

Fig. 5(a) shows the distribution thickness of the ZrO_2_ on the surface of the Z_0.08_, Z_0.08_T_0.2_ and Z_0.08_T_0.3_, which was collected from TEM image Fig. 5(b, c, d). The thickness of coating materials layer was calculated by two software of excel (follow Pythagora's theorem as described in Fig. 5(c)) and directly from ImageJ software. The ZrO_2_-thick is relative uniformity, which varied from 5.06 to 7.08 nm and the average thickness is about 5.75 nm. A ZrO_2_ layer on the surface of Z_0.08_ is varied from 1.42 to 3.96 nm and an average thickness of 2.75 nm. For the Z_0.08_T_0.3_ sample, the thickness of a ZrO_2_ layer varied from 5.9 to 13.4 nm and an average thickness of about 9.68 nm.

### 2.6 Effect of pH value of coating solution on the solubility of the lithium impurities on the surface of Ni-rich layered cathode structure

Fig. 6(a) shows the amount of Li impurities (LiOH/ Li_2_CO_3_) with bare NCM82, washed NCM82, Z_0.08_ and Z_0.08_T_0.2_ sample. The concentration of Li impurities on the surface of the sample is decreased when the amount of TA increases in the coating solution. Videlicet, TA has supported ability dissolution of Li impurities and controlled pH value of coating solution as shown in Fig. 6(b). Concretely, amount of Li impurities were eliminated in a washed sample of (29.02% Li_2_CO_3_, 80.74% LiOH), in Z_0.08_ is (33.7% Li_2_CO_3_, 82.96% LiOH) and in Z_0.08_T_0.2_ is (80.82% Li_2_CO_3_, 85.15% LiOH), compared with the bare sample.

### 2.7 Electrochemical performance

Electrochemical properties of the ZrO_2_-coated NCM82 by different concentration of Zr(SO_4_)_2_ without TA has been reported in a previous paper [Ref]. In this report, the electrochemical performance of Z_0.08_T_x_ (0 ≤ x ≤ 0.4) and bare NCM82 were showed in Fig. 7**,** the half-cells were cycled at 0.3 C after formation cycles 0.2 C and a voltage of 2.5 – 4.25, (1 C = 200 mA *g* ^−^ ^1^). Fig. 7(a) compares the charge/ discharge voltage profiles of the Z_0.08_, Z_0.08_T_0.1_, Z_0.08_T_0.2_, Z_0.08_T_0.3_ and Z_0.08_T_0.4_ samples at 0.2 C and a cut-off voltage of 2.5–4.25 V. Although Z_0.08_T_0.1_ shows the highest specific capacity of 196.8 mAh *g*^−1^ for the initial cycle, it shows poor cycle ability. The Z_0.08_T_0.2_ sample shows the highest capacity retention of 89.35% after 100 cycles and 76.69% after 190 cycles. The Z_0.08_T_0.2_ sample is also shown good cyclability at 45 °C and higher rate performance compared with bare NCM82 (Fig. 7(c, d)).

## Declaration of Competing Interests

The authors declare that they have no known competing financial interests or personal relationships which have, or could be perceived to have, influenced the work reported in this article.

## Appendix Supplementary materials

Image, application 1

Image, application 2
